# Balancing metabolism and reproduction

**DOI:** 10.7554/eLife.97601

**Published:** 2024-04-11

**Authors:** Songdou Zhang, Shiheng An

**Affiliations:** 1 Department of Entomology, China Agricultural University Beijing China; 2 Department of Entomology, Henan Agricultural University Zhengzhou China

**Keywords:** Diaphorina citri, Candidatus Liberibacter asiaticus, Citrus huanglongbing, mutualism, reproduction, metabolism, Other

## Abstract

The bacterium responsible for a disease that infects citrus plants across Asia facilitates its own proliferation by increasing the fecundity of its host insect.

**Related research article** Li J, Holford P, Beattie GAC, Wu S, He J, Tan S, Wang D, He Y, Cen Y, Nian X. 2024. Adipokinetic hormone signaling mediates the fecundity of Diaphorina citri infected by ‘Candidatus Liberibacter asiaticus’. *eLife* **13**:RP93450. doi: 10.7554/eLife.93450.

Citrus huanglongbing – a disease known as ‘citrus cancer’ because of the devastating effect it can have on citrus plants – is caused by a bacterium called *Candidatus* Liberibacter asiaticus (*C*Las; Killiny, 2022). In Asia, this bacterium is primarily spread by a lice-like bug called *Diaphorina citri*. However, it is not possible to study *C*Las in the laboratory because efforts to culture it have been unsuccessful. Therefore, the most effective approach for preventing outbreaks of huanglongbing is to manage the population of *D. citri* (Wang et al., 2017).

Relationships between insects and plant pathogens – like that between *D. citri* and *C*Las – are common in the natural world, and many of these are mutually beneficial to both parties, though some are not (Eigenbrode et al., 2018). In a mutualistic or symbiotic relationship, the pathogen relies on the insect to help it spread from plant to plant, while infection by the pathogen can benefit the insect through an increase in fitness. To date, most research in this area has focused on relationships in which the pathogen is a virus (see, for example, Liu et al., 2014 on the spread of barley yellow dwarf virus by aphids, and Mao et al., 2019 on the spread of ice gall dwarf virus by leafhoppers), so less is known about mutualistic relationships involving bacteria.

Previous studies have revealed a mutualistic relationship in which *C*Las infection boosts the fitness of *D. citri* by increasing its ability to produce large numbers of offspring (fecundity), but the molecular dynamics driving this interaction were not fully understood (Pelz-Stelinski and Killiny, 2016; Ren et al., 2016). Now, in eLife, Yijing Cen, Xiaoge Nian and colleagues from South China Agricultural University, Shaoguan University, and Western Sydney University – including Jiayun Li as first author – report results that shed light on this interaction at the molecular level (Li et al., 2024).

Reproductive development in insects demands a significant energy supply (Yang et al., 2024), so exploring how *C*Las infection regulates and mobilizes energy metabolism in *D. citri* to improve fecundity is a promising avenue to explore. Insects store energy and fat as triglyceride and glycogen, and a process called AKH signaling (where AKH is short for adipokinetic hormone) has a crucial role in converting the former to diglyceride in order to release lipids, and the latter to trehalose to release energy (Arrese and Soulages, 2010).

First, Li et al. demonstrated that *C*Las infection significantly increases levels of triglyceride and glycogen, as well as lipid droplet size. These findings suggest that *C*Las-infected *D. citri* possess greater energy reserves and greater potential for energy mobilization, which may support the development of ovaries in females. Knocking down the gene for AKH (or its receptor) disrupted the mobilization of fat and this resulted in delayed ovary development, decreased egg production, and a reduction in the titer of *C*Las in the ovaries. These findings underscore the critical role of AKH and its receptor in managing the balance between energy metabolism and fecundity in *D. citri*.

To investigate how AKH signaling is regulated, Li et al. identified microRNAs that could potentially bind to AKH mRNA and prevent it being translated into protein. In vivo and in vitro experiments showed that a microRNA called miR-34 directly targets the 3’-untranslated region of the AKH receptor, therefore reducing the levels of this mRNA and the AKH receptor itself. Expression levels of miR-34 were lower in infected *D*. citri than in controls, suggesting that *C*Las may decrease levels of miR-34. Furthermore, treating infected *D. citri* with a synthetic microRNA that mimics the function of miR-34 led to outcomes similar to those observed when the gene for the AKH receptor was knocked down: delayed ovarian development, reduced egg production, and decreased levels of *C*Las in the ovaries (Figure 1). This finding represents the first instance of a host microRNA influencing AKH signaling to impact lipid metabolism and fecundity in infected *D. citri*. Additionally, the research points to an array of genes affected by the AKH signaling cascade, such as those associated with juvenile hormone signaling and two reproduction-related proteins called vitellogenin and vitellogenin receptor.

**Figure 1.**
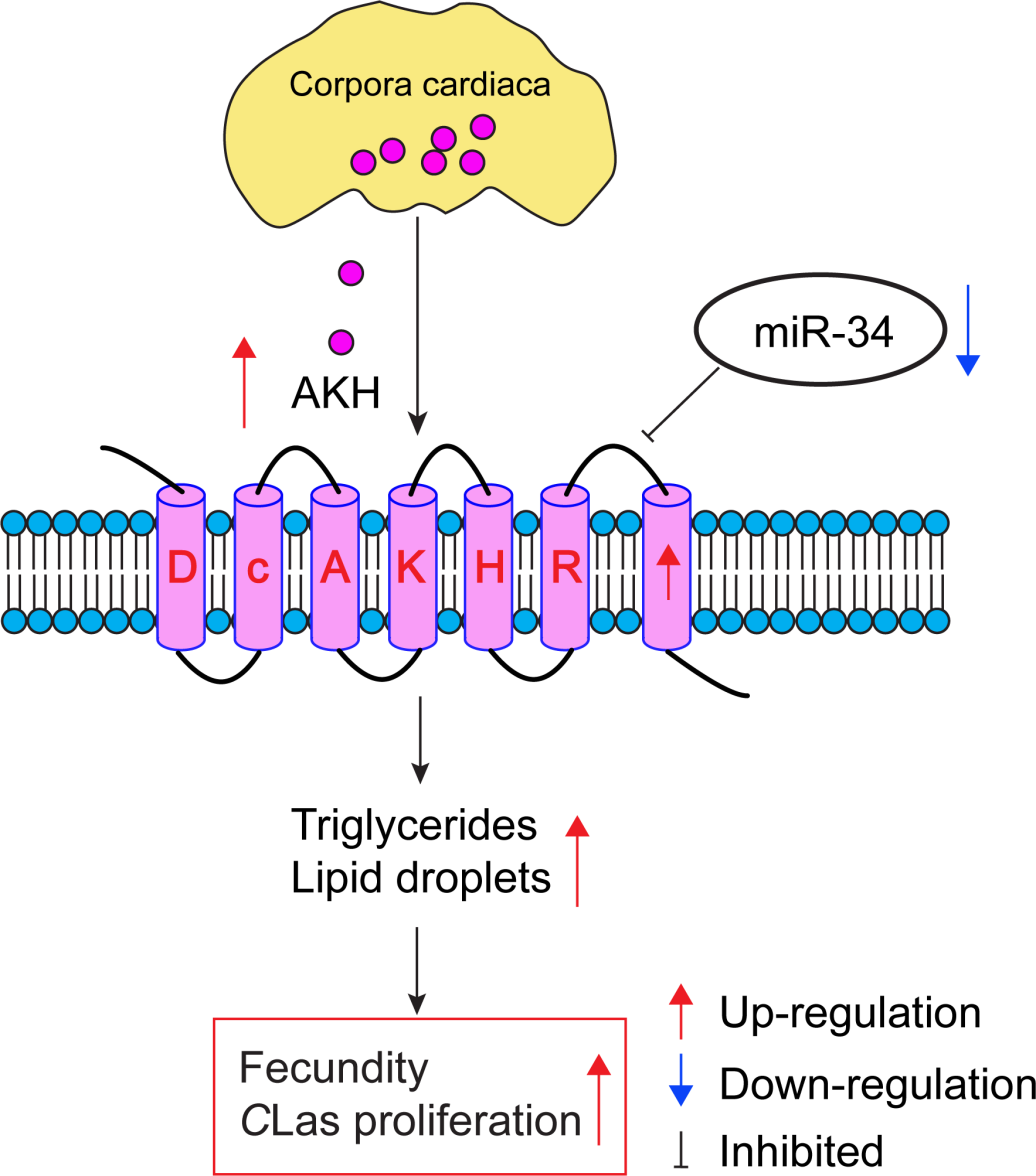
How infection by bacteria can modulate metabolism and increase reproduction in an insect. When the lice-like insect *D. citri* is infected with *C*Las, the bacterium that causes huanglongbing in citrus plants, there is an increase in the production of adipokinetic hormone (AKH; pink circles; top) in the corpora cardiaca (yellow), which is part of the endocrine system of the insect. The AKH molecules bind to AKH receptors (pale pink barrel shapes), and the resulting increase in AKH receptor signaling leads to increased levels of triglyceride and increased numbers of lipid droplets (not shown). A microRNA called miR-34 usually inhibits AKH receptor signaling, but infection with *C*Las also results in the downregulation of miR-34. The increase in AKH receptor signaling caused by *C*Las infection increases the energy reserves available for reproduction, leading to increased fecundity for *D. citri*, which also aids the proliferation of *C*Las. *C*Las: *Candidatus* Liberibacter asiaticus.

The work of Li et al. in highlighting the role of AKH signaling in increasing the fecundity of *D. citri* that have been infected by *C*Las contributes to our understanding of the rapid spread of huanglongbing in the field. Future research should focus on elucidating the intricate interactions among endocrine signals such as hormones, neuropeptides and neurotransmitters, and their collective influence on the increased fecundity prompted by *C*Las infection. Undertaking these investigations will broaden our understanding of the complex relationships between plant pathogens and their insect vectors, and could potentially lead to new methods to control plant pathogen populations.
